# The cost of total hip arthroplasty: compare the hospitalization costs of national centralized procurement and national volume-based procurement

**DOI:** 10.3389/fpubh.2024.1383308

**Published:** 2024-07-08

**Authors:** Yongyong Fan, Qiang Xu, Gang Jin, Lingjun Jiang, Chenglong Wang

**Affiliations:** Department of Orthopaedics, Taizhou Hospital of Zhejiang Province Affiliated to Wenzhou Medical University, Zhejiang, China

**Keywords:** national volume-based procurement (NVBP), national centralized procurement (NCP), implant cost, composition of hospitalization cost, total hip arthroplasty

## Abstract

**Background:**

With the increasing demand for joint replacement surgery in China, the government has successively issued the policies of national centralized procurement (NCP) and national volume-based procurement (NVBP) of artificial joints. The purpose of this study is to evaluate the impact of NCP and NVBP policies on hospitalization cost, rehospitalization and reoperation rate of total hip arthroplasty (THA).

**Methods:**

In total, 347 patients who underwent THA from January 2019 to September 2022 were retrospectively analyzed. According to the implementation of NCP and NVBP, patients were divided into three groups: control group (*n* = 147), NCP group (*n* = 130), and NVBP group (*n* = 70). Patient-level data on the total hospitalization costs, rehospitalization rate, THA reoperation rate and inpatient component costs were collected before and after the implementation of the policies and Consumer Price Index was used to standardize the cost.

**Results:**

After the implementation of NCP and NVBP, the total cost of hospitalization decreased by $817.41 and $3950.60 (*p* < 0.01), respectively. The implantation costs decreased from $5264.29 to $4185.53 and then rapidly to $1143.49 (*p* < 0.01), contributing to increased total cost savings. However, the cost of surgery and rehabilitation increased after NCP and NVBP implementation (*p* < 0.01). The proportion of implants decreased from 66.76 to 59.22% and then to 29.07%, whereas that of drugs increased from 7.98 to 10.11% and then to 12.06%. The proportion of operating expenses rose from 4.86 to 8.01% and then to 18.47%. Univariate linear regression analysis showed that hospital stay, NCP and NVBP were correlated with total hospitalization cost (*p* < 0.01). Multivariate analysis showed that hospital stay, NCP and NVBP were independent predictors of total hospitalization cost (*p* < 0.01).

**Conclusion:**

In this study, hospital stay, NCP, and NVBP were independent predictors of total inpatient costs. After the implementation of NVBP policy, the cost of implants and hospitalization has decreased significantly, and the technical labor value of medical staff has increased, but a multifaceted method is still needed to solve the problem of increasing costs of other consumables. Limitations of the study suggest the need for further and more comprehensive evaluation in the future.

## 1 Introduction

More than one million hip arthroplasty operations are performed each year worldwide (1). The number of initial and revision surgeries has increased year by year throughout history. For example, the total number of hip replacements in the UK grew by 37% between 2008 and 2017 (2), with similar increases reported in Sweden, New Zealand and South Korea (3–5).

At present, many countries around the world are faced with the challenge of increasing medical expenditure (6, 7), with the global pharmaceutical market, which was valued at 955 billion dollars in 2019, being the major contributor (8). In China, total health expenditure increased from 145.4 billion yuan in 2008 to 5.7998 trillion yuan in 2018, with an average annual compound growth rate of 13.4% (9). In 2018, the total pharmaceutical expenditure in China was 1914.89 billion yuan, accounting for 32.39% of the total health expenditure (10), much higher than an average of 17% for the countries of the Organization for Economic Cooperation and Development (11). To curb the growth of medical expenditure and reduce drug costs, several countries have successfully implemented the national centralized drug procurement (NCDP) (12–14). In January 2019, the Chinese government launched a national volume-based procurement (NVBP) policy aimed at reducing drug costs and improving the drug procurement mechanism (15). A key feature of the first round of this policy, also known as “4 + 7” policy, is “price for price” (16). The policy has been successful in reducing price by an average of 52% for 25 winning products, with a price reduction of as high as 96% in some products (17).

Various factors can affect the cost of hospitalization for TJA. Studies have shown that age, hospital stay, and postoperative ICU stay are factors that influence the cost of TKA (18). In addition, patient and hospital characteristics also have an impact on hospitalization costs (19). The growth of China’s economy has been accompanied by a significant increase in regional inequality, including health care disparities. Hospitalization costs are likely to be affected by this disparity in supply-side inputs into the health care system and access to health care for residents (20).

Research has shown that from July 2008 to June 2015, the implant cost of joint replacement in the United States accounted for 46.3% of the internal hospital costs (21). An analysis of TKA hospitalization costs in China’s national database from 2013 to 2019 shows that implants and materials account for about 60–70 percent of total hospitalization costs (19). With the rising demand for joint replacement surgery in China, on November 20, 2020, the Medical Price and Bidding Procurement Guidance Center of the National Medical Security Bureau issued a notice on the rapid collection and price monitoring of the second batch of NCP data of high-value medical consumables, including artificial hip and knee joint prostheses (22). On June 4, 2021, the National Health Insurance Bureau and eight other departments issued guidance on the NVBP and use of high-value medical consumables (23). Its aim was to further reduce the cost of high-value medical consumables. On June 21, 2021, the National Organization Joint Procurement Office of High-value Medical Consumables issued the national organization for the NVBP of artificial joints (No. 1) and launched the National Organization for the NVBP of artificial joints (24). To the best of our knowledge, the impact of NCP and NVBP on various components of inpatient expenditure is unknown, and there is no report on reoperation and rehospitalization rate after implementation of these policies. Therefore, this study evaluated the impact of NCP and NVBP on hospitalization cost, rehospitalization rate and reoperation rate of total hip arthroplasty (THA).

## 2 Materials and methods

This study selected THA patients admitted to our hospital from January 2019 to September 2022. The following inclusion criteria were used: (1) patients who underwent surgery due to osteoarthritis, femoral neck fracture, ischemic necrosis, and dysplasia, (2) all patients who underwent primary unilateral THA, and (3) the joint prostheses used were winning products of NCP or NVBP from the same company. The following exclusion criteria were used: (1) patients diagnosed with infectious arthritis, rheumatoid arthritis or other autoimmune diseases, (2) patients undergoing femoral head replacement or revision surgery, (3) patients using joint prostheses or non-winning products from other companies, (4) patients undergoing additional surgery during hospitalization for other diseases, (5) patients who cannot tolerate surgery due to severe internal and external diseases, and (6) patients with incomplete data. According to the inclusion and exclusion criteria, a total of 347 patients were included in this study and divided into three groups: control group (*n* = 147), NCP group (*n* = 130), and NVBP group (*n* = 70).

### 2.1 Patient and public involvement

Patients or the public were not involved in the design, or conduct, or reporting, or dissemination plans of our research.

### 2.2 Data

After approval by the institutional review committee, all clinical data for the 347 THA patients admitted between January 2019 and September 2022were obtained from the hospital’s inpatient electronic medical record system V6.0 (GOODWILL, Beijing, China). Demographic data such as age, sex, BMI, operation side, and ASA score of all patients, as well as hospitalization expenses, internal component costs, and the rate of rehospitalization and reoperation within 3 months after discharge were collected.

### 2.3 Three research stages

According to the introduction of national policies and the implementation of policies in Zhejiang Province, we divided patients into three groups: (1) control group (from January 2019 to November 19, 2020), (2) NCP group (from November 20, 2020 to May 5, 2022 during the implementation of the second batch of NCP policy of national high-value medical consumables) (22), and (3) NVBP group (from May 05 to September 2022). The implementation of NVBP policy of artificial joint prosthesis was in Zhejiang Province on May 5, 2022 (25).

### 2.4 Hospitalization expenses and categories

**Figure 1:**
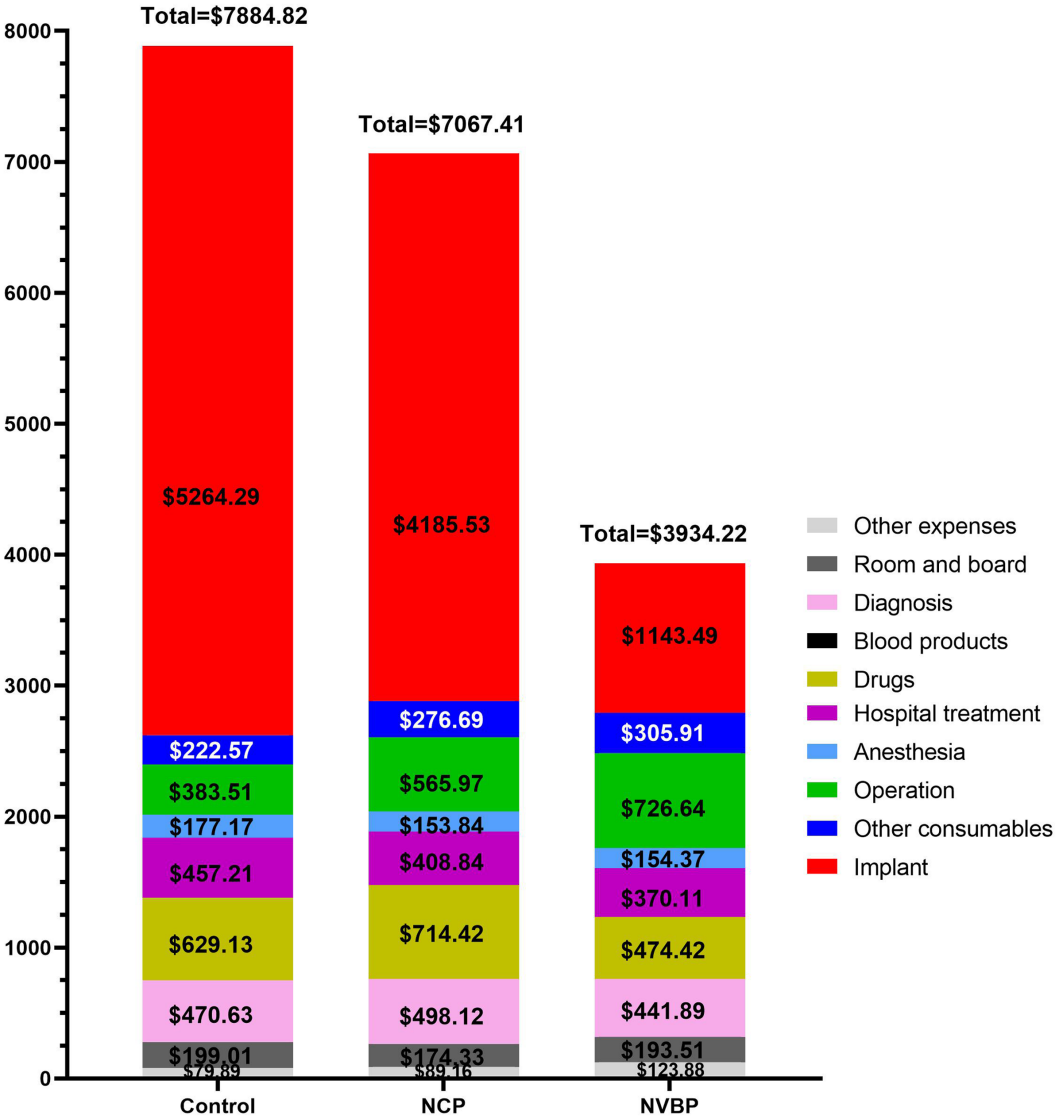
A bar chart showing the total hospitalization costs for three groups of THA patients and costs for all major categories. NCP, national centralized procurement; NVBP, national volume-based procurement.

We classified the expenses during hospitalization into 10 categories: (1) implant; (2) other consumables; (3) operation; (4) anesthesia; (5) hospital treatment; (6) drugs; (7) blood products; (8) diagnosis; (9) room and board; and (10) other expenses (Figure 1). Among them, the hospitalization expenses included comprehensive treatment, traditional Chinese medicine treatment, rehabilitation and nursing. The cost of diagnosis included laboratory, imaging, clinical and pathology. The overall and partial savings were calculated according to the internal costs of the hospital.

By using the Consumer Price Index (26), all expenditure variables were adjusted for inflation and economic growth to reflect the real dollar value (2022). After that, all values were converted into US dollars (US$) using the average exchange rate (26) of US$1 = 6.7434 (from 2019 to 2022).

### 2.5 Statistical analysis

All data were analyzed using SPSS26.0 (IBM, Armonk, New York, USA). The measurement data and count data are statistically described using the mean plus or minus standard deviation (X ± SD), and frequency and percentage, respectively. Kruskal-Wallis one-way analysis of variance by ranks was used to compare the differences among three independent samples and between the two groups. Chi-square test was used to compare categorical variables. Univariate linear regression analysis was used to evaluate the relationship between each factor and the total hospitalization cost. Then, the factors with significant differences in univariate analysis and other possible factors were analyzed by multiple linear regression analysis to determine the independent predictors of total hospitalization cost. *p* < 0.05 indicates statistical significance.

## 3 Results

### 3.1 General information

A total of 347 patients were included in this study, including 147 in the control group. They had an average age and BMI of 61.58 years (SD ± 10.05, range 29–80 years) and 23.49 kg/m^2^ (SD ± 3.22), respectively, and included 74 males (50.3%), 73 females (49.7%), with 86 right hips (58.5%) and 61 left hips (41.5%). The NCP group included 130 patients comprising 70 males (53.8%) and 60 females (46.2%), with an average age and BMI of 63.09 years (SD ± 10.98, range 30–82 years) and 23.89 (SD ± 3.42), respectively, and 71 right hips (54.6%) and 59 left hips (45.4%). The NVBP group had 70 cases comprising 31 males (44.3%) and 39 females (55.7%), with an average age and BMI of 64.16 years (SD ± 10.69, range 31–88 years) and 23.52 (SD ± 3.22), respectively, and 34 right hips (48.6%) and 36 left hips (51.4%). There was no difference in the demographic variables among the three groups (Table 1).

**Table 1:** Demographic data.

Parameter	Control (*n* = 147)	NCP (*n* = 130)	NVBP (*n* = 70)	*p* value
Age(y)	61.58 ± 10.05(29–80)	63.09 ± 10.98(30–82)	64.16 ± 10.69(31–88)	0.134
Gender				
Male	74(50.3%)	70(53.8%)	31(44.3%)	0.435
Female	73(49.7%)	60(46.2%)	39(55.7%)	
Operation side				
Right	86(58.5%)	71(54.6%)	34(48.6%)	0.386
Left	61(41.5%)	59(45.4%)	36(51.4%)	
BMI (kg/m^2^)	23.49 ± 3.22	23.89 ± 3.42	23.52 ± 3.22	0.564
ASA				
2	136(92.5%)	113(86.9%)	60(85.7%)	0.201
3	11(7.5%)	17(13.1%)	10(14.3%)	

NCP, national centralized procurement; NVBP, national volume-based procurement; ASA, American Society of Anesthesiologists; BMI, body mass index.

### 3.2 Quality of care

There were no statistically significant changes in readmission or reoperation in each of the three groups. From January 2019 to September 2022, the control group, NCP group and NVBP group had an average readmission rate of 3.40, 3.85 and 2.86% and reoperation rates of 2.72, 2.31 and 1.43%, respectively (Table 2).

**Table 2:** Comparison of reoperation and readmission among the three groups.

Parameter	Control (*n* = 147)	NCP (*n* = 130)	NVBP (*n* = 70)	*p* value
Readmission	5(3.40%)	5(3.85%)	2(2.86%)	0.934
reoperation	4(2.72%)	3(2.31%)	1(1.43%)	0.839

NCP, national centralized procurement; NVBP, national volume-based procurement.

### 3.3 Total hospitalization costs and internal component costs

**Figure 2:**
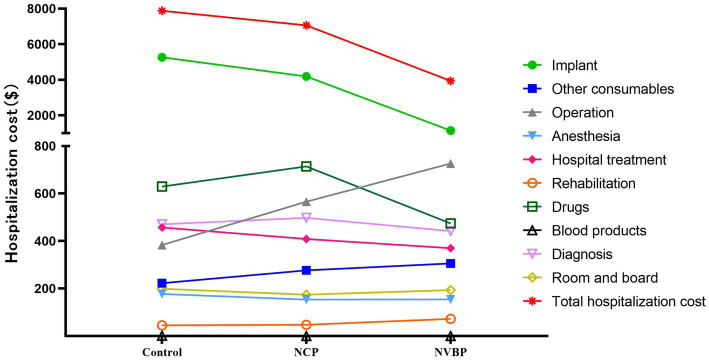
A line chart showing the total costs of hospitalization for three groups of THA patients and the cost trend for all major categories. NCP, national centralized procurement; NVBP, national volume-based procurement.

The total cost of hospitalization for THA patients decreased by $817.41 (from $7884.82 to $7067.41) after the implementation of NCP policy compared with $3950.60 (from $7884.82 to $3934.22) after the implementation of NVBP policy, the total cost of hospitalization decreased, with a statistically significant difference between the groups (*p* < 0.01). During the hospitalization period, the total cost savings were mainly contributed by lower implantation costs. These costs decreased from $5264.29 to $4185.53, and then rapidly to $1143.49, with a statistically significant difference between the groups (*p* < 0.01). The cost of surgery and rehabilitation significantly increased after the implementation of the policies (*p* < 0.01). Other consumables also increased after the implementation of NCP policy (*p* < 0.01), but there was no significant difference between the NCP and NVBP groups (*p* = 0.835). The cost of drugs increased after the implementation of the NCP policy, but decreased significantly after the implementation of the NVBP policy (*p* < 0.01) (Figure 2; Tables 3, 4).

**Table 3:** Comparison of total hospitalization cost and internal component cost among three groups.

Parameter	Control (*n* = 147)		NCP (*n* = 130)		NVBP (*n* = 70)		*p* value
	x̅±S	95%CI	x̅±S	95%CI	x̅±S	95%CI	
Hospital stay	9.93 ± 4.20	9.25, 10.62	7.56 ± 3.15	7.01, 8.11	6.36 ± 2.38	5.79, 6.93	0.000
Implant	5264.29 ± 130.14	5243.08, 5285.50	4185.53 ± 112.11	4166.07, 4204.98	1143.49 ± 61.53	1128.82, 1158.16	0.000
Other consumables	222.57 ± 80.88	209.38, 235.75	276.69 ± 50.52	267.92, 285.45	305.91 ± 102.04	281.58, 330.24	0.000
Operation	383.51 ± 4.36	382.80, 384.22	565.97 ± 128.16	543.73, 588.21	726.64 ± 0.00	726.64, 726.64	0.000
Anesthesia	177.17 ± 17.94	174.24, 180.09	153.84 ± 22.49	149.94, 157.74	154.37 ± 23.32	148.81, 159.93	0.000
Hospital treatment	457.21 ± 179.93	427.88, 486.54	408.84 ± 208.60	372.64, 445.04	370.11 ± 137.73	337.27, 402.95	0.000
Rehabilitation	45.32 ± 45.67	37.87, 52.76	47.31 ± 20.00	43.84, 50.78	72.25 ± 28.90	65.36, 79.14	0.000
Drugs	629.13 ± 234.33	590.94, 667.33	714.42 ± 265.06	668.43, 760.42	474.42 ± 148.77	438.94, 509.89	0.000
Blood products	1.40 ± 9.77	−0.19, 3.00	0.51 ± 5.84	−0.50, 1.52	0.00 ± 0.00	0.00, 0.00	0.365
Diagnosis	470.63 ± 135.87	448.48, 492.78	498.12 ± 148.38	472.38, 523.87	441.89 ± 117.68	413.83, 469.95	0.020
Room and board	199.01 ± 112.49	180.67, 217.35	174.33 ± 81.87	160.12, 188.54	193.51 ± 88.20	172.48, 214.54	0.166
Total hospitalization cost	7884.82 ± 605.86	7786.06, 7983.58	7067.41 ± 578.88	6966.96, 7167.86	3934.22 ± 457.01	3825.24, 4043.19	0.000

All values are in US dollars (US$).

NCP, national centralized procurement; NVBP, national volume-based procurement; CI, confidence interval.

**Table 4:** Pairwise comparison results among the three groups.

Parameter	NCP-Control	NVBP-Control	NCP- NVBP
Hospital stay	0.000	0.000	0.004
Implant	0.000	0.000	0.000
Other consumables	0.000	0.000	0.835
Operation	0.000	0.000	0.000
Anesthesia	0.000	0.000	0.801
Hospital treatment	0.004	0.000	0.253
Rehabilitation	0.000	0.000	0.000
Drugs	0.004	0.000	0.000
Diagnosis	0.203	0.072	0.005
Total hospitalization cost	0.000	0.000	0.000

NCP, national centralized procurement; NVBP, national volume-based procurement.

### 3.4 Change of internal composition ratio of hospitalization expenses

**Figure 3:**
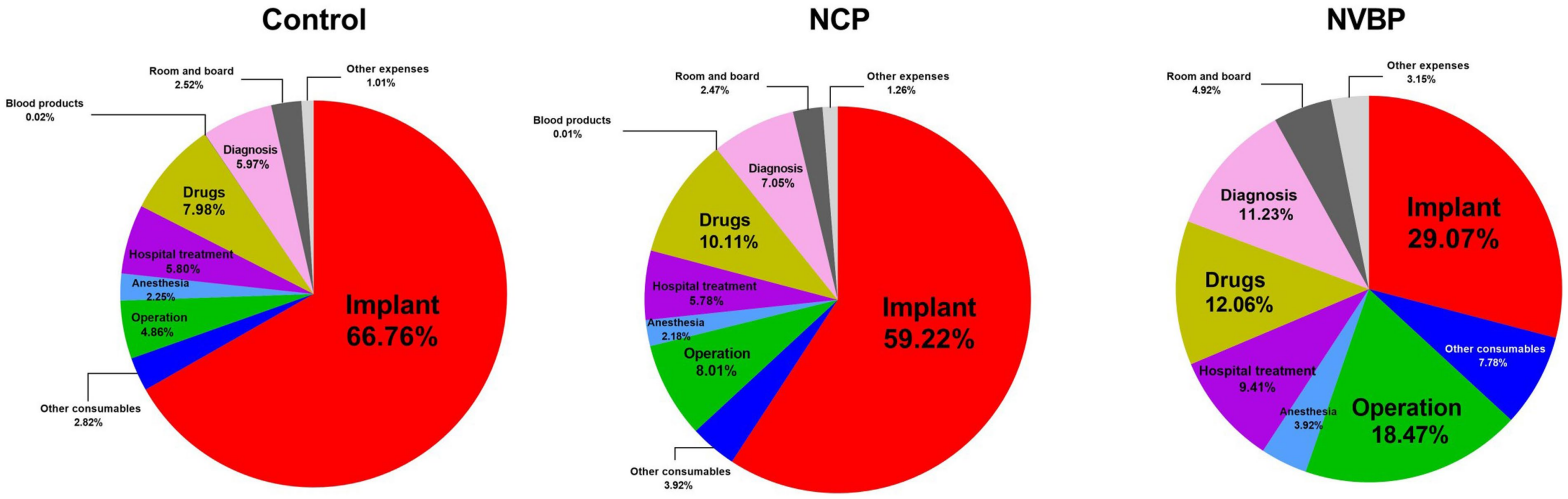
A pie chart showing the percentage of each component of the total hospital cost for three groups of THA patients. NCP, national centralized procurement; NVBP, national volume-based procurement.

Before the implementation of the policy, implants and drugs accounted for 66.76 and 7.98% of the total hospitalization expenses of patients, respectively. The other items accounted for less than 6%. After the implementation of NCP policy, the proportion of implants decreased to 59.22%, whereas the proportion of drugs and operating expenses increased to 10.11% and from 4.86 to 8.01%, respectively. After the implementation of the NVBP policy, the change of internal composition ratio was significant. The proportion of implants decreased to 29.07%, whereas the proportion of drugs and surgical expenses increased to 12.06 and 18.47%, respectively (Figure 3; Supplementary Table 1).

### 3.5 Hospital savings by changes in internal cost

During the NCP period, the reduction in implantation costs accounted for most of the hospital’s total savings. Cost savings from implants were $1078.76, accounting for 91.73% of the total savings, with a saving rate of 20.49%. The total cost of hospitalization decreased by $817.41 or 10.37% but the cost of other consumables and operation increased by $54.12 or 24.32% and $182.46 or 47.58%, respectively. After the implementation of the NVBP policy, cost savings from the implants were $4120.80, accounting for 93.21% of the total savings, with a saving rate of 78.28%. The total cost of hospitalization decreased by $3950.60 or 50.10% but the cost of other consumables and operation increased by $83.34 or 37.44% and $343.13 or 89.47%, respectively (Table 5).

**Table 5:** Total and within component savings by internal hospital cost.

Parameter	Control (*n* = 147)	NCP (*n* = 130)	Savings	Total savings in NCP, %	Savings within component, %	NVBP (*n* = 70)	Savings	Total savings in NVBP, %	Savings within component, %
Implant	5264.29	4185.53	1078.76	91.73	20.49	1143.49	4120.80	93.21	78.28
Other consumables	222.57	276.69	−54.12		−24.32	305.91	−83.34		−37.44
Operation	383.51	565.97	−182.46		−47.58	726.64	−343.13		−89.47
Anesthesia	177.17	153.84	23.33	1.98	13.17	154.37	22.80	0.52	12.87
Hospital treatment	457.21	408.84	48.37	4.11	10.58	370.11	87.10	1.97	19.05
Drugs	629.13	714.42	−85.29		−13.56	474.42	154.71	3.50	24.59
Blood products	1.40	0.51	0.89	0.08	63.57	0.00	1.40	0.03	100.00
Diagnosis	470.63	498.12	−27.49		−5.84	441.89	28.74	0.65	6.11
Room and board	199.01	174.33	24.68	2.10	12.40	193.51	5.50	0.12	2.76
Other expenses	79.89	89.16	−9.27		−11.60	123.88	−43.99		−55.06
Total	7884.82	7067.41	817.41		10.37	3934.22	3950.60		50.10

All values are in US dollars (US$).

NCP, national centralized procurement; NVBP, national volume-based procurement.

### 3.6 Analysis of influencing factors of total hospitalization cost in THA patients

Univariate linear regression analysis showed that hospital stay, NCP and NVBP were the main influencing factors of total hospitalization cost (*p* < 0.01). And there were no significant differences in Age, Gender, Operation side, BMI and ASA (*p* > 0.05) (Table 6).

**Table 6:** Univariate linear regression with total hospitalization cost as dependent variable.

Parameter	*B*	SE	*β*	*t*	*p*	95%CI	*F*	Adjusted R^2^
Age	3.898	8.079	0.026	0.483	0.630	−11.991, 19.788	0.233	−0.002
Gender	170.228	170.089	0.054	1.001	0.318	−164.314, 504.770	1.002	0.000
Operation side	−119.273	171.082	−0.038	−0.697	0.486	−455.768, 217.221	0.486	−0.001
BMI	−21.603	25.885	−0.045	−0.835	0.405	−72.515, 29.309	0.697	−0.001
ASA	−42.299	272.711	−0.008	−0.155	0.877	−578.684, 494.087	0.024	−0.003
Hospital stay	235.016	18.604	0.562	12.632	0.000	198.424, 271.608	159.578	0.314
NCP	456.983	174.221	0.140	2.623	0.009	114.315, 799.651	6.880	0.017
NVBP	−3566.982	90.337	−0.905	−39.485	0.000	−3744.664, −3389.301	1559.073	0.818

*B*, unstandardized regression coefficients; SE, standard error; *β*, standardized regression coefficients; CI, confidence interval; VIF, Variance inflation factor; BMI, body mass index; ASA, American Society of Anesthesiologists; NCP, national centralized procurement; NVBP, national volume-based procurement.

The results of multiple linear regression analysis showed that the regression equation was significant (*F* = 1790.087, *p* < 0.001). Among them, age (*β* = 0.049, *p* < 0.001) and hospital stay (*β* = 0.296, *p* < 0.001) significantly positively predicted total hospitalization cost, NCP (*β* = −0.164, *p* < 0.001) and NVBP (*β* = −0.895, *p* < 0.001) significantly negatively predicted total hospitalization cost. Together, these variables explained 95.4% of the variation in total hospitalization cost (Table 7).

**Table 7:** Multiple linear regression with total hospitalization cost as dependent variable.

Parameter	*B*	SE	*β*	*t*	*p*	95%CI	*F*	Adjusted R^2^	VIF
Age	7.296	1.780	0.049	4.098	0.000	3.794, 10.798	1790.087	0.954	1.056
Hospital stay	123.556	5.340	0.296	23.139	0.000	113.053, 134.058			1.225
NCP	−535.571	43.120	−0.164	−12.420	0.000	−620.386, −450.757			1.306
NVBP	−3527.728	53.506	−0.895	−65.932	0.000	−3632.969, −3422.486			1.382

*B*, unstandardized regression coefficients; SE, standard error; *β*, standardized regression coefficients; CI, confidence interval; VIF, Variance inflation factor; NCP, national centralized procurement; NVBP, national volume-based procurement.

## 4 Discussion

As part of the medical reforms implemented in China in recent years, NCP and NVBP have played an important role in reducing drug prices and promoting rational use of drugs (27–30). Wang et al. (31) showed that the implementation of the “4 + 7” policy increased the use of cardiovascular generic drugs and significantly improved access to affordable drug costs for patients. Wen et al. (32) showed that batch procurement successfully reduced the price of selective serotonin reuptake inhibitors and improved access to affordable drugs, especially for patients with chronic diseases. Since its implementation, NVBP has successfully reshaped the Chinese drug market structure, making it more conducive to high-quality and low-cost generic drugs. With its success in reducing drug prices, the next focus of the NVBP policy is to decrease the high cost of medical consumables. On July 3, 2020, the Medical Price and Bidding Guidance Center of the National Medical Security Bureau issued the national organization coronary stent NVBP Scheme (Draft for Comments) (33). This marked the beginning of NVBP of high-value medical consumables in the country. On November 5, 2020, the NVBP of high-value medical consumables was held in Tianjin, which lead to a price reduction from about 13,000 yuan to about 700 yuan for coronary stents. With the successful lowering of the price of coronary stents, similar NCP and NVBP initiatives have been launched for artificial hip and knee joint prostheses (22–24).

In this study, we analyzed the impact of NCP and NVBP policies on hospitalization costs, rehospitalization and reoperation rate of THA patients. After the implementation of the NCP policy, the total cost of hospitalization decreased by 10.37%, from $7884.82 to $7067.41. This cost decreased further by 50.10%, reaching $3934.22 after the implementation of the NVBP policy (Table 5). Thus, NCP shows a direct effect on reducing the inpatient burden.

A large part of the total cost savings of hospitals was contributed by lower implant costs. After the implementation of the NCP and NVBP policies, the cost of implants decreased by 20.49 and 78.28%, accounted for 91.73 and 93.21% of the total hospital savings, respectively (Table 5). This finding is particularly important as it highlights the advantages of NCP and NVBP policies in China, especially in reducing the burden of hospitalization for patients.

In addition to the significant decline in the cost of implants, the internal constituent ratio of patients in hospital has changed greatly. However, the proportion of implants decreased significantly, whereas the cost of drugs, surgery, and other consumables, as well as hospitalization costs, only changed to a lesser extent (Figure 3; Supplementary Table 1). During the implementation of the two policies, the operating expenses increased by 89.47%, first from $383.51 to $565.97 and then to $726.64 (Table 5). Similarly, the proportion of the total expenses increased from 4.86 to 18.47% (Figure 3; Supplementary Table 1). Overall, the hospital treatment increased by 19.05%. The increase in operation and treatment costs reflects the increase in technical labor value of medical staff. However, these costs did not contribute to the decrease in total hospitalization cost. This shows that the implementation of NVBP policy has had a positive impact on the control of the total cost of hip joint consumables and helped shape the structure of medical expenses. It is suggested that the medical insurance department should properly raise the price of joint surgery under the condition of comprehensive consideration of surgical labor cost, technical difficulty, risk factors and the affordable range of medical insurance fund, so as to reflect the value of medical personnel’s technical labor.

The rehabilitation cost increased steadily from $45.32 to $72.25, whereas the length of hospitalization decreased (Table 3). The increase in rehabilitation costs suggests that there is a greater focus on interventions to improve the physical function of patients after surgery. There was no significant increase in readmission and reoperation rate of patients (*p* > 0.05), suggesting that the reduction in hospital stay did not affect the quality of medical services (Table 2).

This study found that whereas the price of implants decreased after the implementation of the policies, that of other consumables increased by 37.44%, reaching $83.34 (Table 5). The consumables included medical 3 M film, surgical suture and disposable anesthetic consumables. As a manager of medical institutions, it is necessary to further strengthen the use of clinical consumables after the implementation of NVBP policy, and guide clinical departments to rationally use medical consumables, especially other consumables except those necessary for surgery, so that NVBP policy can better benefit patients. Reforms in the management of high-value medical consumables take time and require cooperation among multiple departments. For successful reforms, the medical insurance, finance, health care and other departments must actively generate and share their experiences and continue to explore and improve the centralized procurement of various high-value consumables to ultimately reduce the medical burden of patients.

Several previous studies have summarized possible influencing factors for TJA hospitalization costs and reported that patient characteristics, age, hospital stay, postoperative ICU stay, hospital characteristics, socioeconomic factors, and healthcare system factors were associated with hospitalization costs (18–20). Similarly, our study showed that age was associated with hospitalization costs, where hospital stay, NCP, and NVBP policies were independent predictors of hospitalization costs.

Our research has several limitations. First, the sample size was small, and therefore, more data are needed to make strong statistical analysis of the results. Second, the study did not analyze the impact of confounding factors such as socioeconomic factors and health care system factors on hospitalization costs, which could increase bias. The study assessed patients’ total hospital costs, which were not covered by Medicare, so the impact of health care system factors was relatively small. Third, the study’s quality of care metrics are limited to rehospitalization and reoperation rates, which could neglect patient-reported outcomes, complication rates, and other clinical measures essential for a comprehensive assessment. Finally, the study was conducted in a single hospital in China, so the findings might not be generalizable to other healthcare settings, especially considering the diverse economic backgrounds of different provinces/cities in China.

## 5 Conclusion

This study confirmed that Hospital stay, NCP, and NVBP were independent predictors of total hospitalization costs for THA patients. After the implementation of the NVBP policy, the hospitalization expenses incurred by patients decreased significantly, mainly due to the lower cost of implants. The cost of operation and treatment increased, suggesting that an improvement in the technical labor value of the medical personnel. However, the cost of other consumables continued to increase. The implementation of NVBP policy has a positive impact on controlling the cost of consumables for total hip joint. However, a multifaceted method is still needed to solve the problem of increasing costs of other consumables, so as to further reduce the medical burden of patients and shape the structure of medical expenses. Further research in the future will require larger, more diverse samples, longer follow-up periods, and a more comprehensive assessment of costs and the quality of care outcomes.

## Data availability statement

The raw data supporting the conclusions of this article will be made available by the authors, without undue reservation.

## Ethics statement

The studies involving humans were approved by the Ethics Committee of Taizhou Hospital of Zhejiang Province, affiliated to Wenzhou Medical University. The studies were conducted in accordance with the local legislation and institutional requirements. Written informed consent for participation was not required from the participants or the participants’ legal guardians/next of kin in accordance with the national legislation and institutional requirements.

## Author contributions

YF: Conceptualization, Data curation, Software, Writing – original draft. QX: Investigation, Software, Writing – original draft. GJ: Data curation, Writing – original draft, Investigation. LJ: Conceptualization, Data curation, Investigation, Writing – original draft. CW: Conceptualization, Data curation, Investigation, Methodology, Software, Writing – original draft, Writing – review & editing.
