# Supplementary material for: The cost of total hip arthroplasty: compare the hospitalization costs of national centralized procurement and national volume-based procurement
Source: Front Public Health. 2024 Jul 8;12:1383308. doi: 10.3389/fpubh.2024.1383308 (PMC11260701; doi:10.3389/fpubh.2024.1383308)
Supplement: Supplementary file 1 [file Table_1.docx]

Supplementary table 1 Proportion of total hospitalization cost and internal component cost among three groups

| Parameter | Control (n=147) | Proportion (%) | NCP (n=130) | Proportion (%) | NVBP (n=70) | Proportion (%) |
| --- | --- | --- | --- | --- | --- | --- |
| Implant | 5264.29 | 66.76 | 4185.53 | 59.22 | 1143.49 | 29.07 |
| Other consumables | 222.57 | 2.82 | 276.69 | 3.92 | 305.91 | 7.78 |
| Operation | 383.51 | 4.86 | 565.97 | 8.01 | 726.64 | 18.47 |
| Anesthesia | 177.17 | 2.25 | 153.84 | 2.18 | 154.37 | 3.92 |
| Hospital treatment | 457.21 | 5.80 | 408.84 | 5.78 | 370.11 | 9.41 |
| Drugs | 629.13 | 7.98 | 714.42 | 10.11 | 474.42 | 12.06 |
| Blood products | 1.40 | 0.02 | 0.51 | 0.01 | 0.00 | 0.00 |
| Diagnosis | 470.63 | 5.97 | 498.12 | 7.05 | 441.89 | 11.23 |
| Room and board | 199.01 | 2.52 | 174.33 | 2.47 | 193.51 | 4.92 |
| Other expenses | 79.89 | 1.01 | 89.16 | 1.26 | 123.88 | 3.15 |
| Total | 7884.82 |  | 7067.41 |  | 3934.22 |  |

All values are in US dollars (US$).

NCP, national centralized procurement; NVBP, national volume-based procurement
